# Flavonoids from *Lycium barbarum* leaves attenuate obesity through modulating glycolipid levels, oxidative stress, and gut bacterial composition in high-fat diet-fed mice

**DOI:** 10.3389/fnut.2022.972794

**Published:** 2022-07-28

**Authors:** JiaLe Liao, Jia Guo, YinHong Niu, Tian Fang, FangZhou Wang, YanLi Fan

**Affiliations:** ^1^Department of Food Science and Technology, School of Food & Wine, Ningxia University, Yinchuan, China; ^2^Ningxia Red Power Goji Co., Ltd., Zhongwei, China; ^3^Ningxia Engineering Research Center for Goji Biological Fermentation & Milling, Zhongwei, China

**Keywords:** *Lycium barbarum* leaves, flavonoids, obesity, oxidative stress, gut bacteria

## Abstract

Traditional herbal therapy made from *Lycium barbarum* leaves has been said to be effective in treating metabolic diseases, while its exact processes are yet unknown. Natural flavonoids are considered as a secure and reliable method for treating obesity. We thus made an effort to investigate the processes by which flavonoids from *L. barbarum* leaves (LBLF) reduce obesity. To assess the effectiveness of the intervention following intragastric injection of various dosages of LBLF (50, 100, and 200 mg/kg⋅bw), obese model mice developed via a high-fat diet were utilized. Treatment for LBLF may decrease body weight gain, Lee’s index, serum lipids levels, oxidative stress levels, and hepatic lipids levels. It may also enhance fecal lipids excretion and improve glucose tolerance. Additionally, LBLF therapy significantly restored gut dysfunction brought on by a high-fat diet by boosting gut bacterial diversities and altering the composition of the gut bacterial community by elevating probiotics and reducing harmful bacteria.

## Introduction

Obesity is becoming one of the biggest global dangers to public health and quality of life due to its rising prevalence (1). Obesity is a significant risk factor for metabolic illnesses and is closely related to our ways of life, dietary habits, environment, and genetic factors (2, 3). The overconsumption of a high-calorie diet dominated by high fat and saccharides causing an imbalance between energy intake and expenditure, thereby driving excessive fat accumulation as triglycerides (TG) in white adipose cells takes primarily charge of the development of obesity (4, 5). When obesity first develops, high blood pressure, low-grade inflammation, non-alcoholic fatty liver, hyperlipidemia, insulin resistance, and cancer are also frequently present (6-8).

Weight-reducing medications like orlistat have recently been thought of as one of the traditional methods for managing obesity. However, they have significant side effects such as increased gastrointestinal exhaust, greasy stools, diarrhea, and hepatotoxicity (9, 10). Given that their characteristics are safe, effective, and have few side effects, active components from the plant kingdom must be found to build functional foods or novel anti-obesity medications. As a well-known traditional medicine, *Lycium barbarum* leaves have a variety of tonic benefits on people, including tonifying liver and kidney, improving eyesight, and removing toxic heat. Recent research found that *L. barbarum* leaves’ abundance of nutrients and natural bioactive substances, such as polysaccharides, flavonoids, minerals, alkaloids, vitamins, and trace elements, significantly improved immunological function, promoted cell proliferation, and decreased blood glucose levels (11, 12).

It has been determined how high oxidative stress promotes the etiology of obesity. Increasing cellular oxidative stress levels will permanently damage macromolecules, including lipids, DNA, and proteins, impairing cellular transport and metabolism and eventually triggering the development of chronic illnesses (13, 14). In contrast, excessive fat storage may boost reactive oxygen species (ROS), causing systemic oxidative stress by raising endoplasmic reticulum stress and NADPH oxidase activity in adipocytes (15). Therefore, obesity is a result of oxidative stress as well as a risk factor for it. However, studies found that flavonoids greatly improved antioxidant capacity in rats fed a high-fat diet (16) and reduced the generation of ROS in cells (17). Another important regulating aspect in the development of obesity is the makeup of the gut microorganisms, which are thought of as a “new organ” at this time (18). Growing attention has recently been given to the roles that gut microorganisms play in the pathogenesis of metabolic disorders and how bioactive dietary ingredients reduce obesity by changing the makeup and functionality of intestinal microbes. Studies have shown that flavonoids might alter the composition of the gut bacterial population in conjunction with increased probiotics and diminished harmful bacteria, lowering the formation of endotoxins and enhancing intestinal barrier function (19). Gut microbial dysbiosis is frequently caused by aberrant changes in host homeostasis (20), which raises the risk of metabolic diseases. In light of these findings, gut microorganisms in conjunction with bioactive dietary ingredients can be considered a cutting-edge method of treating obesity.

There are currently few publications on the effectiveness of *L. barbarum* leaves extract in treating obesity. Thus, the effects of the LBLF supplement were studied in mice fed a high-fat diet better to understand the processes behind LBLF’s anti-obesity properties.

## Materials and methods

### Extraction and purification of LBLF

The dried *L. barbarum* leaves (Yinchuan Yuxin Co., Ltd., Yinchuan, China) were crushed and sieved into uniform powder size. The powder (1 g) was mixed with 70 volumes of 70% ethanol (*v/v*) solution and extracted two times at 70°C (2 h each time). The extraction was filtered using a vacuum pump (SHZ-III, Shanghai, China) and then merged. The ethanol was recycled by rotary evaporation (Re-52AA, Shanghai, China) at 55°C to collect the extract. Next, the petroleum ether was used to remove chlorophyll and lipids in the extract. Finally, the petroleum ether was recycled by rotary evaporation to collect the ultimate crude extract. The crude flavonoids were obtained by vacuum freeze-drying (JDG-0.2, Lanzhou, China).

The 0.375 g of the crude flavonoids powder and 2 g of D101 macroporous resin (Shanghai Yuanye Biotechnology Co., Ltd., Shanghai, China) were put into a 250 ml triangular bottle containing 100 ml ultrapure water. Then, the triangular bottle is fixed on the shaking table to oscillate at room temperature for 24 h. Under the same experimental conditions, then filtered and dried macroporous resin (2 g) was mixed with 100 ml of 70% ethanol (*v/v*) solution so that flavonoids could be eluted. The eluent was evaporated, concentrated, and freeze-dried to collect the flavonoid product. The total flavonoid content was measured by Al(NO_3_)_3_-NaNO_2_ colorimetric using rutin (≥98%) as reference (21).

### Experimental animals and groups

Healthy male ICR mice, weighing 26 ± 1 g and aging 6–7 weeks, were purchased from the Experimental Animal Centre of Ningxia Medical University (animals certificate number: SYXK 2020-0001). All animal experiments were permitted by the Animal Welfare and Ethics Committee of Ningxia Medical University (No. IACUC-NYLAC-2020-179). During the experiment, the animals were housed in a controlled environment (SPF) with a humidity range of 40–70%, a temperature range of 20–25°C, and a light and dark cycle for 12 h. All animals could be free to obtain food and water. After 1 week of acclimatization, the mice (a total of 40 animals) were randomly divided into two groups: the normal control group (NC) and the obese model construction group (OMC). Without intragastric administration of LBLF, OMC’s mice were given a high-fat diet for 30 days. After fasting for 14 h, blood was taken from the orbit by a capillary tube to detect serum lipids. If there was a notable difference in serum levels of TC, TG, HDL-C, and LDL-C between the NC and OMC groups, it indicated that the model was successfully established. Afterward, the OMC’s mice were randomly divided into four groups (eight animals per group): high-fat diet group (HFD), low-dosage group of 50 mg/kg⋅bw LBLF (LBLFL), medium-dosage group of 100 mg/kg⋅bw LBLF (LBLFM) and a high-dosage group of 200 mg/kg⋅bw LBLF (LBLFH). In the next 8 weeks, the mice in LBLFL, LBLFM, and LBLFH groups were gavage-given different doses of total flavonoids of *L. barbarum* leaves at 2:00 p.m., respectively. Daily diet provision: NC group given basic diet, HFD and LBLF-dosage groups given a high-fat diet. High-fat diet (Shuangshi Co., Ltd., Suzhou, China) formula contained basic diet 58.0%, casein 8.4%, lard 20.4%, sucrose 7%, sodium cholate 1.3% and cholesterol 1.6% (w/w). The collected blood was centrifuged at 3,500 rpm/min for 10 min to obtain serum. All tissue samples and feces were stored at −20°C.

### Serum biochemical analysis as well as hepatic and fecal lipids measurement

The serum alanine aminotransferase (ALT), aspartate aminotransferase (AST), total cholesterol (TC), triglyceride (TG), low-density lipoprotein cholesterol (LDL-C), and high-density lipoprotein cholesterol (HDL-C) levels were assayed with AU400 Clinical Biochemistry Analyzer (Olympus Co., Ltd., Tokyo, Japan). The serum lipase (LPS) and non-esterified fatty acid (NEFA) levels were assayed by reagent kits (Nanjing Jiancheng Co., Ltd., Nanjing, China). The contents of triglyceride (TG) and cholesterol (TC) in the liver and feces were also assayed by reagent kits (Nanjing Jiancheng Co., Ltd., Nanjing, China). The improved soxhlet extraction method was used to measure feces’ total fat (TF).

### Oral glucose tolerance test

After fasting for 10 h, the respective blood was collected from tail veins at 0, 30, 90, and 120 min after orally giving glucose (2.0 g/kg⋅bw) and then measured with a Sannuo glucometer and glucose strips (22). Finally, drawing the plot of glucose concentration with time aimed to obtain the areas under each curve (AUC).

### Detection of oxidative stress markers

The serum and liver activity of catalase (CAT), glutathione peroxidase (GSH-P_*x*_) and superoxide dismutase (SOD), contents of malondialdehyde (MDA) and protein carbonyl (PC), and total antioxidant capacity (T-AOC) and quantitative protein were assayed by reagent kits (Nanjing Jiancheng Co., Ltd., Nanjing, China).

### Histopathological analysis

Fresh liver and abdominal adipose tissue were fixed with 10% paraformaldehyde, embedded in paraffin wax after gradient dehydration, sliced into 5 μm sections, and then stained with hematoxylin and eosin. Finally, morphological characteristics of the tissue sections were observed by microscope (Motic Co., Ltd., China).

### Analysis of intestinal bacterial composition

The total genomic DNA of the fecal samples was acquired by DNA extraction kit (Omega Engineering Inc., United States). Using 1% agarose gel electrophoresis to detect DNA concentrations, the DNA was diluted to 1 ng/μl using Milli-Q water. The barcoded primers hyper-variable V3-V4 region of the 16S rRNA gene of the diluted DNA was amplified by a primer combination of 515F (5’-GTGCCAGCMGCCGCGGTAA-3’), and 806R (5’-GGACTACHVGGTWTCTAAT-3’). All PCR reactions were operated in a total volume of 50 μl reaction system including 0.2 μM of forward and reverse primers, 15 μl of Phusion High-Fidelity PCR Master Mix, and approximately 10 ng template DNA, as well as PCR-grade water, was used to adjust the volume. The use of Qiagen Gel Extraction Kit (Qiagen Co., Ltd., CA, United States) to purify mixture PCR products. All the samples were sequenced by NovaSeq 6000 (Illumina Co., Ltd., San Diego, CA, United States).

### Statistical analysis

All data were expressed as means ± SD. The multiple comparisons were analyzed with LSD or Duncan test using one-way ANOVA analysis, while the paired comparisons were carried out by *t*-test. *p*-value < 0.05 (* or #) exhibited a significant difference and *p* < 0.01 (^**^ or ##) exhibited an extremely significant difference. All figures were drawn with Graphpad Prism 8.0 or origin 2021 tools. Spearman’s method was used for correlation analysis using origin 2021.

## Results

### The establishment of obese mice model after 30 days of high-fat diet intervention

As shown in Table 1, the OMC group saw significant increases in body weight and Lee’s index relative to the NC group (*p* < 0.05). Meanwhile, the serum levels of TG, TC, and LDL-C were noticeably elevated, whereas the serum level of HDL-C noticeably decreased (*p* < 0.05). These findings showed that obese mouse models with abnormalities of lipids metabolism were effectively developed.

**TABLE 1:** Body weight, Lee’s index, and serum biochemical markers on the 30th day.

Parameters	NC	OMC
Body weight (g)	39.03 ± 1.26	42.39 ± 2.79^#^
Lee’s index	324.01 ± 6.78	330.53 ± 5.79^#^
TC (mmol/L)	3.92 ± 0.34	8.51 ± 1.24^##^
TG (mmol/L)	0.68 ± 0.07	1.14 ± 0.06^#^
HDL-C (mmol/L)	2.59 ± 0.17	1.24 ± 0.31^##^
LDL-C (mmol/L)	0.79 ± 0.08	1.31 ± 0.17^#^

NC, normal control group; OMC, obese model construction group.

^#^p < 0.05 and ^##^p < 0.01 vs. NC group.

### Effects of LBLF on the food intake, water intake, body weight increase, and Lee’s index

**FIGURE 1:**
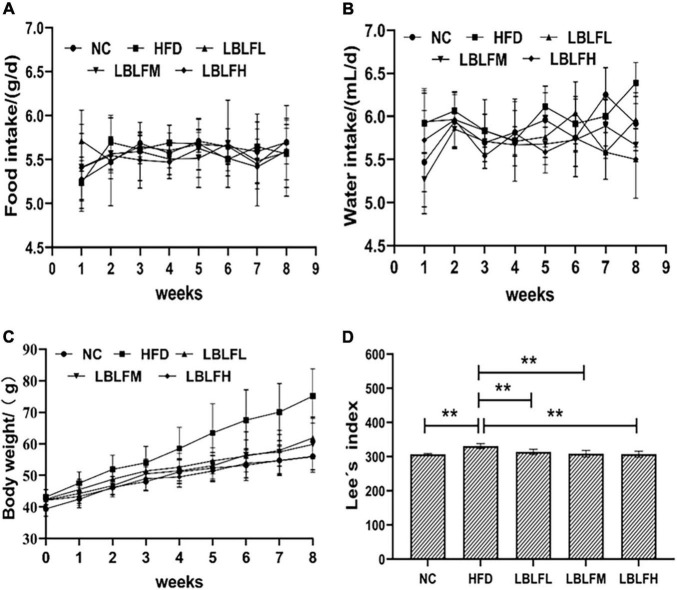
Effects of LBLF supplement on **(A)** food intake, **(B)** water intake, **(C)** body weight, and **(D)** Lee’s index in obese mice. (*p < 0.05) and (**p < 0.01) vs. HFD group.

The HFD group saw the most pronounced increases in body weight, as seen in Figure 1. In contrast to the NC group, the HFD and LBLFL groups showed an apparent rise in body weight after 8 weeks of treatment with LBLF (*p* < 0.05), while the LBLFM and LBLFH groups exhibited no significant difference (Figure 1C) (*p* > 0.05). Apparently, Lee’s index was significantly increased in the HFD group, which was statistically different from the other groups (Figure 1D) (*p* < 0.01). Lee’s index in the LBLFL group showed a notable rise that was statistically different (*p* < 0.05) from the NC group, whereas that of the LBLFM and LBLFH groups exhibited no remarkable difference (Figure 1D) (*p* > 0.05).

### Effects of LBLF on serum biochemical markers, hepatic lipids, and oral glucose tolerance in obese mice

As shown in Table 2, in contrast to the NC group, serum TC, TG, LDL-C, AST, ALT, NEFA, and blood glucose levels in the HFD group showed a significant rise, while the serum HDL-C level showed a marked drop (*p* < 0.01). However, LBLF administration, especially for the medium and high dosages of LBLF, caused a lower serum AST, ALT, TG, TC, LDL-C, NEFA, blood glucose levels, and a higher serum level of HDL-C than the HFD group, and the intervention efficacy of LBLF treatment groups was similar (*p* < 0.05 or *p* < 0.01).

**TABLE 2:** Serum biochemical markers and hepatic lipids levels after 12 weeks.

Sample	Parameters	NC	HFD	LBLFL (50 mg/kg⋅bw)	LBLFM (100 mg/kg⋅bw)	LBLFH (200 mg/kg⋅bw)
Serum	TC (mmol/L)	4.89 ± 0.46**	9.65 ± 0.35^##^	9.21 ± 0.44^##^	7.16 ± 0.62^##^*	6.21 ± 0.51^#^**
	TG (mmol/L)	0.95 ± 0.08**	2.14 ± 0.19^##^	1.82 ± 0.36^##^	1.56 ± 0.11^##^**	1.41 ± 0.21^#^**
	HDL-C (mmol/L)	3.69 ± 0.17**	1.89 ± 0.22^##^	2.52 ± 0.64^##^*	2.75 ± 0.22^##^*	3.33 ± 0.16**
	LDL-C (mmol/L)	0.82 ± 0.34**	1.97 ± 0.14^##^	1.54 ± 0.19^##^*	1.37 ± 0.24^#^*	1.02 ± 0.24**
	Glucose (mmol/L)	3.7 ± 0.29**	8.1 ± 0.32^##^	7.4 ± 0.77^##^*	5.4 ± 0.68^##^**	4.8 ± 0.49^##^**
	NEFA (μmol/L)	276.42 ± 47.79**	657.20 ± 61.11^##^	548.52 ± 50.87^##^*	527.48 ± 56.77^##^*	456.51 ± 59.82^##^**
	AST (U/L)	97.23 ± 9.96**	186.87 ± 11.26^##^	165.33 ± 7.18^##^*	142.65 ± 8.91^##^**	122.77 ± 12.89^#^**
	ALT (U/L)	41.73 ± 5.99**	116.37 ± 10.11^##^	107.47 ± 10.49^##^	84.33 ± 5.72^##^**	61.17 ± 3.94^#^**
Liver	TC (μmol/g liver wt)	13.65 ± 3.10**	41.82 ± 7.36^##^	35.66 ± 5.12^##^	28.85 ± 5.98^##^*	20.58 ± 4.67**
	TG (μmol/g liver wt)	33.59 ± 3.31**	65.57 ± 4.62^##^	63.88 ± 6.69^##^	53.82 ± 3.78^##^**	44.36 ± 3.17^#^**

^#^p < 0.05 and ^##^p < 0.01 vs. NC group; *p < 0.05 and ^**^p < 0.01 vs. HFD group.

Table 2 indicated that hepatic lipids, including TG and TC, in the HFD group were higher than the in NC group with remarkable differences (*p* < 0.01). However, LBLF administration, especially for the medium and high dosages of LBLF, caused a remarkable reduction of TC and TG in comparison with the HFD group (*p* < 0.05), and a more remarkable reduction was shown in the high doses of the LBLF group (*p* < 0.01).

**FIGURE 2:**
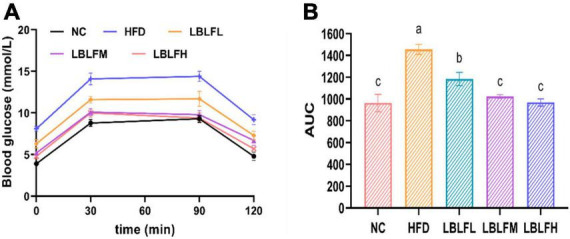
Effects of LBLF supplement on glucose tolerance in obese mice. **(A)** Oral glucose tolerance test (OGTT); **(B)** the integrated areas under each curve (AUC) after oral glucose. Different lowercase letters with significant differences (*p* < 0.05).

Figure 2 showed that the AUC of blood glucose concentration with time in the HFD group was notably increased (*p* < 0.05) in contrast with the NC group. However, the AUC in the LBLF-dose groups was notably reduced (*p* < 0.05) in contrast to the HFD group.

### The regulation of LBLF on content of fecal lipids and activity of serum lipase in obese mice

**FIGURE 3:**
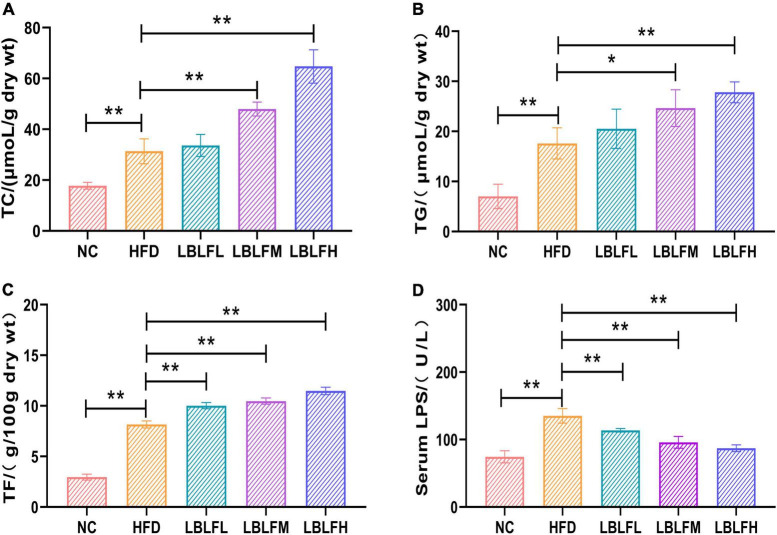
Effects of LBLF supplement on fecal lipids content and serum LPS (lipase) activity in obese mice. **(A)** Mean content of TC (total cholesterol); **(B)** mean content of TG (total triglyceride); **(C)** mean content of TF (total fat); **(D)** mean activity of serum LPS (lipase). (*p < 0.05) and (**p < 0.01) vs. HFD group.

Figure 3 showed the findings from a measurement of the lipids, including TC, TG, and TF, in feces. The HFD group had higher TC, TG, and TF contents, which was statistically different (*p* < 0.01) from the NC group. However, in contrast to the HFD group, LBLF treatment, particularly for the medium and high doses of LBLF, induced a noticeable elevation of TC, TG, and TF in feces (Figures 3A–C). Furthermore, with higher LBLF administration, serum LPS activity in LBLF-dose groups was decreased (*p* < 0.01) (Figure 3D). The findings mentioned above showed that LBLF intervention could successfully raise fat contents in feces.

### LBLF suppresses hepatic and serous oxidative stress in obese mice

**FIGURE 4:**
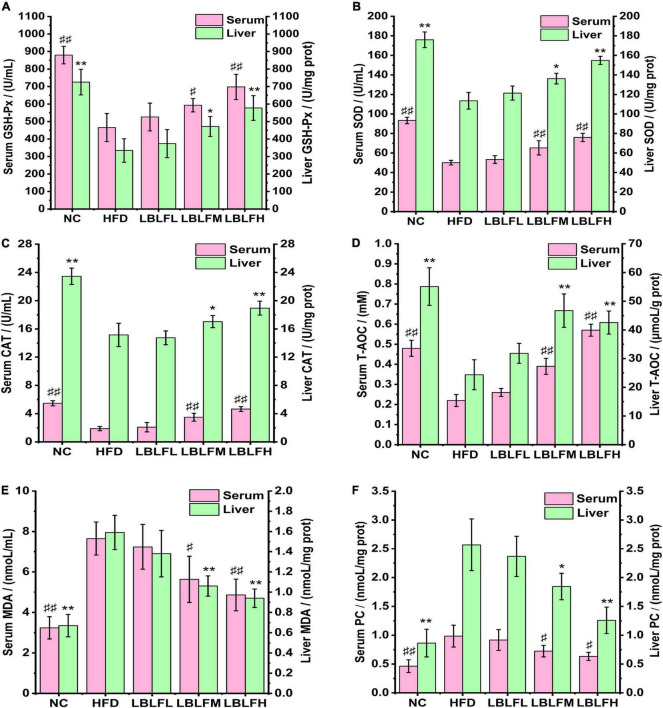
Effects of LBLF supplement on markers of oxidative stress, including **(A)** GSH-P_*x*_, **(B)** SOD, **(C)** CAT, **(D)** T-AOC, **(E)** MDA, and **(F)** PC, in serum and liver. Serum markers: (^#^*p* < 0.05) and (^##^*p*<0.01) vs. HFD group. Liver markers: (*p < 0.05) and (** *p* < 0.01) vs. HFD group.

The serous and hepatic antioxidant enzyme activities (GSH-P_*x*_, CAT, SOD), MDA, PC contents, and T-AOC were measured to evaluate oxidative stress levels in mice and the results are seen in Figure 4. In contrast to the NC group, serum GSH-P_*x*_, CAT, SOD activities, and T-AOC in the HFD group were remarkably reduced (*p* < 0.01), while MDA and PC contents were remarkably increased (*p* < 0.01), which was almost the same variations in those of liver. In contrast with the HFD group, serum GSH-P_*x*_, SOD, and CAT activities, and T-AOC in medium and high doses of LBLF exhibited a remarkable rise (*p* < 0.01 or *p* < 0.05), while MDA and PC contents showed a notable drop (*p* < 0.01 or *p* < 0.05), which was similar alterations in those of liver.

### Pathological analysis of liver and adipose tissues

**FIGURE 5:**
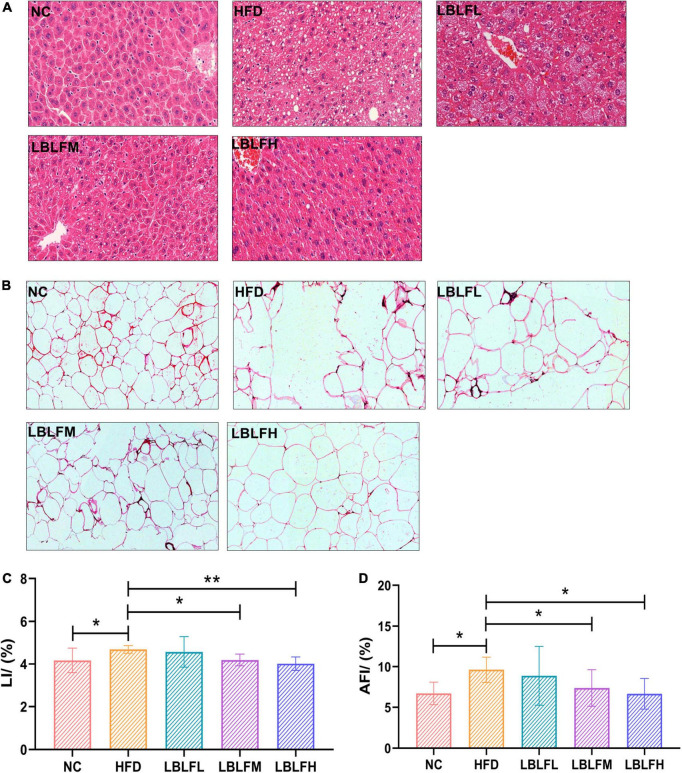
Effects of LBLF supplement on photographs of HE-stained sections of tissues and their related indexes in obese mice. **(A)** HE-stained sections of liver (×200); **(B)** HE-stained sections of abdominal fat tissues (×200); **(C)** LI (liver index); **(D)** AFI (abdominal fat index). (*p < 0.05) and (**p < 0.01) vs. HFD group.

As shown in Figure 5, HE staining revealed that the adipocytes in the HFD group were inhomogeneous in size and figure. Moreover, other histopathological features including cellular rupture, fusion, and denaturation also appeared in the HFD group (Figure 5B). Obviously, high-fat diet-induced multiple histopathological symptoms were effectively lightened after administration with LBLF. In contrast to the NC group, hepatocytes filled with a considerable number of varying sizes of fat vacuoles were observed in the HFD group. Nevertheless, the number of adipocytes showed a notable reduction in the liver tissues of the LBLFM and LBLFH groups compared to the HFD group (Figure 5A). High-fat diet intervention caused a notable increase in LI (Figure 5C) and AFI (Figure 5D) (*p* < 0.05), while the LI and AFI showed a noticeable reduction with the increased LBLF dosages and the significant differences of the LBLFM and LBLFH groups were found (*p* < 0.05) from the HFD group.

### Effects of LBLF on the gut bacterial composition in obese mice

**FIGURE 6:**
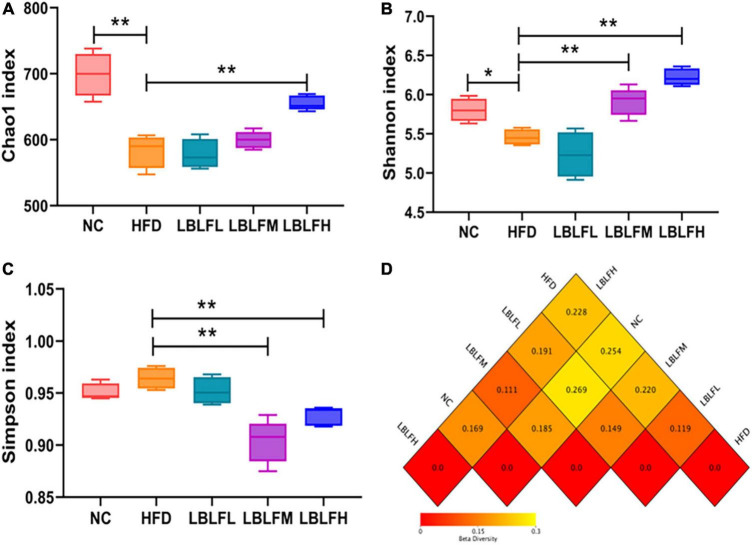
Effects of LBLF supplement on gut microbial α-diversity and β-diversity in obese mice. **(A)** Chao1 index, **(B)** Shannon index, and **(C)** Simpson index as the estimators of α-diversity of gut microbiota. Increased α-diversity with the increase of Chao1 index and Shannon index as well as the reduction of Simpson index. **(D)** Distance matrix heatmap as the estimators of β-diversity of gut microbes showed the extent of the similarities of the intestinal microbial compositions. A smaller difference coefficient means a bigger extent of the similarity (*p < 0.05) and (**p < 0.01) vs. HFD group.

Figure 6 illustrates indices linked to α-diversities to display the variety of gut bacteria. Additionally, a heatmap of the β-diversity analysis by distance matrix was employed to provide a profile of the extent of the similarities of the intestinal bacterial compositions across groups. High-fat diet intervention resulted in a more pronounced loss in the diversity of the bacterial community than in the NC group, according to the α-diversities related indexes (Figures 6A–C). Nevertheless, after LBLF therapy, the gut bacterial diversities were gradually restored. Additionally, LBLF administration enhanced the extent of similarity of microbial community compositions between LBLF treatment groups and NC groups with decreased difference coefficient on heatmap (Figure 6D), according to the β-diversity analysis.

**FIGURE 7:**
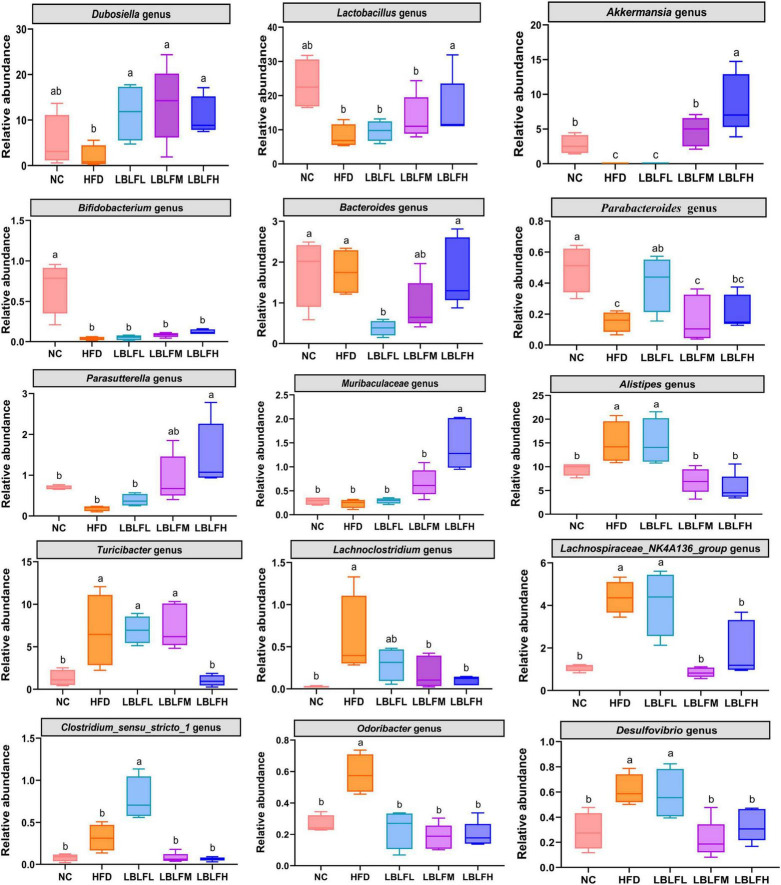
Comparison of relative abundances of the 15 vital intestinal microbes at the genus level. Different lowercase letters with significant differences (*p* < 0.05).

As shown in Figure 7, higher relative abundances of *Lachnoclostridium*, *Alistipes*, *Lachnospiraceae_NK4A136*_*group*, *Desulfovibrio*, *Odoribacter*, *Turicibacter*, and lower relative abundances of *Dubosiella*, *Lactobacillus*, *Akkermansia*, *Parasutterella*, *Bifidobacterium*, and *Parabacteroides* were exhibited in the HFD group than the NC group, while LBLF administration partially reversed these changes. The relative contents of the remaining three microbial genera including *Bacteroides*, *Clostridium_sensu_stricto_1*, and Muribaculaceae in the HFD group exhibited no obvious difference in contrast with the NC group. Furthermore, relative abundances of *Bacteroides* and *Muribaculaceae* were obviously increased concerning the supplement of LBLF. Meanwhile, the relative content of *Clostridium_sensu_stricto_1* was obviously reduced with increased LBLF dosages.

**FIGURE 8:**
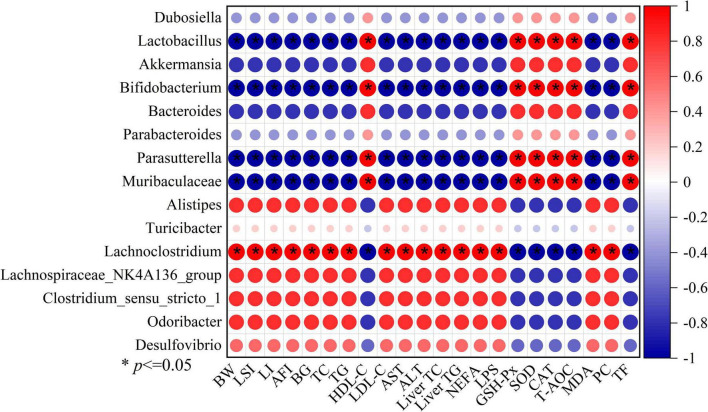
Spearman’s correlation analysis between 15 key intestinal microbes and 22 obesity-related indexes, including BW (body weight), LSI (Lee’s index), LI (liver index), AFI (abdominal fat index), BG (blood glucose), serum TC, TG, HDL-C, LDL-C, AST, ALT, liver TC, liver TG, NEFA (serum non-esterified fatty acids), LPS (serum lipase), serum GSH-P_*x*_, SOD,CAT, T-AOC, MDA, PC (protein carbonyl), and TF (total fecal fat). Significant correlations with (* *p* < 0.05).

Additionally, we used Spearman’s correlation analysis to determine the relationships between changes in filtered 15 key microbial genera and high-fat diet-related indexes (the body weight, Lee’s index, liver index, abdominal fat index, serum lipids level, serum transaminase levels, hepatic lipids levels, blood glucose levels, and oxidative stress levels) in order to investigate the impact of gut microbial compositions on the high-fat diet-induced obesity (Figure 8). The relative abundances of seven microbes, including *Lachnoclostridium*, *Alistipes*, *Lachnospiraceae_NK4A136*_*group*, *Desulfovibrio*, *Odoribacter*, *Clostridium_sensu_stricto_1*, and the *Turicibacter*, were positively correlated with high-fat diet-associated indexes. Instead, the relative abundances of 6 microbes that benefit from LBLF, including *Dubosiella*, *Lactobacillus*, *Akkermansia*, *Parasutterella*, *Bifidobacterium*, *Bacteroides*, and Muribaculaceae, were inversely correlated with high-fat diet-associated indexes.

## Discussion

One heterocyclic ring (C) and two phenolic rings (A and B) make up the fundamental chemical structure of flavonoids, and hundreds of other compounds may be defined by modifying this structure (23). The anti-obesity, anti-hyperlipidemia, hepatoprotective, and antioxidative properties of plant-derived flavonoids have been demonstrated to offer significant therapeutic promise (24, 25). Our previous research detected five phenolic monomers (rutin, chlorogenic acid, kaempferol, coumaric acid, and caffeic acid) from total flavonoid extract in *L. barbarum* leaves (26). In addition, the content of LBLF was detected by Al(NO_3_)_3_-NaNO_2_ colorimetric, and exceeded 83% in this study. Experimental animals on a high-fat diet frequently develop intestinal bacterial disorders in addition to an abnormal rise in body weight, serum lipids levels, and blood glucose levels (27). Through a high-fat diet intervention, we successfully created obese model mice with excessive body weight and lipids metabolism abnormalities. In high-fat diet-induced rats with hyperlipidemia, flavonoids were shown to lower serum TG, TC, LDL-C, AST, and ALT levels as well as body weight (28), which was consistent with our findings. After being exposed to a high-fat diet, the liver—one of the primary metabolic organs *in vivo*—deposited too many adipose tissues, damaging hepatocytes and then raising the risk of obesity (29). As anticipated, LBLF supplementation effectively attenuated fatty liver and its related metabolic alterations in obese mice.

We discovered that LBLF significantly raised the amount of TC, TG, and TF in feces while successfully inhibiting serum LPS activity. According to growing evidence, the primary enzyme for digesting lipids, pancreatic lipase, may hydrolyze approximately between 50 and 70% of all ingested fat in the intestinal tract (30). Some of the findings revealed that natural products, particularly flavonoids, might be a class of important inhibitors of pancreatic lipase (31, 32). For instance, Hou et al. (33) revealed that *in vitro* pancreatic lipase activity was strongly inhibited by flavonoids from Cortex Mori Radicis. We previously looked at the potent inhibitory effects of LBLF on pancreatic lipase *in vitro*, with a half-inhibitory concentration (IC_50_) of (0.910 ± 0.008) mg/ml, and reversible and non-competitive inhibition as the kind of inhibition (34). We might therefore conclude that LBLF’s inhibitory impact on pancreatic lipase activity, similar to the effects of weight-loss medications like orlistat, may be responsible for the increased fecal fat contents.

Increased oxidative stress was caused by a high-calorie diet and high dietary saturated fatty acids by stimulating multiple intracellular mechanisms including decreased antioxidant defenses, NADPH oxidases mediated superoxide production, oxidative phosphorylation in mitochondria, glycoxidation, protein kinase C, chronic inflammation, and postprandial ROS generation, etc (35). High-level oxidative stress could stimulate the excessive deposition of adipose tissues, thereby accelerating the development of obesity by promoting the pre-adipocytes proliferation and increase of differentiated adipocytes in size (36, 37). However, natural flavonoids were responsible for ameliorating oxidative stress via stimulating enzyme activities or participating in several signaling pathways associated with oxidative stress *in vivo*, which had been fully elucidated in previous research (38, 39).

The antioxidant enzymes (GSH-P_*x*_, CAT, and SOD) as one of the principal members of the antioxidant defense system effectively relieved oxidative damage (40). The GSH-P_*x*_ and SOD are mainly responsible for scavenging lipids peroxides such as MDA, blocking the chain reaction of free radicals. The superoxide ion free radicals can effectively be decomposed into hydrogen peroxide under SOD actions. The CAT can decompose harmful hydrogen peroxide. Hu et al. (41) demonstrated that flavonoids could improve antioxidant capacity via the increase of gene expression levels related to antioxidation such as SOD-3.

Flavonoids’ outstanding antioxidant abilities seem to be primarily attributed to their structural traits, which include their metal-chelating properties, the stereochemical features of the molecule, and their hydrogen donating properties of hydroxyl groups in ring B (42, 43). By chelating pro-oxidant metal ions like Cu(II), Zn(II), and Fe(II), for instance, flavonoids prevented the formation of ROS and free radicals, thereby blocking the process of lipids peroxidation and proteins oxidation (44), which may explain the reduction of MDA and protein carbonyl, one of the final markers of lipids and proteins peroxidation, respectively, caused by LBLF. Additionally, flavonoids’ hydrogen-donating abilities prevented free radical chain reactions. HDL-C was reported to exert an anti-atherogenic effect through reversely transporting cholesterol, but it was easily oxidized due to its excellent antioxidative actions (45). It is clear that LBLF can shield HDL-C from oxidative deterioration. Thus, using flavonoids to prevent the harm caused by free radicals and oxidants may be a successful therapeutic strategy for treating obesity.

Correlation analysis between intestinal bacteria and high-fat diet-associated indices in our study indicated that gut microecology, a crucial internal environmental element, had significant effects on the development of obesity. In addition to improving antioxidant capacity and increasing fecal lipids excretion, *Lactobacillus*, *Bifidobacterium*, *Akkermansia*, *Muribaculaceae*, and *Parasutterella* also decreased serum and hepatic lipids levels. Instead, *Turicibacter*, *Lachnoclostridium*, *Alistipes*, *Lachnospiraceae_NK4A136*_*group*, *Desulfovibrio*, and *Odoribacter* significantly exacerbated the detrimental metabolic alterations in a high-fat diet. By producing metabolites and enzymes such as bile acids, indole, short-chain fatty acids, and caseinolytic proteas B as signal molecules, gut microorganisms may interact with the intestinal barrier’s operation and other metabolic processes (46, 47). LBLF intervention obviously increased the relative content of *Dubosiella* genus, which was convinced to be a potential gut beneficial bacterium due to its anti-inflammatory and anti-oxidation properties. Researchers discovered that by increasing the activity of bile brine hydrolysis enzymes, *Lactobacillus* and *Bifidobacterium* might encourage the breakdown of hepatic lipids and enhance the excretion of fecal lipids (48). Numerous studies showed that *Lactobacillus* and *Bifidobacterium* enhanced the generation of tryptophan-derived metabolites, which were directly related to the body weight loss in rats and the reduction of liver damage via effects on numerous essential tryptophan catabolic enzymes (49, 50). Additionally, by promoting the growth of epithelial cells and the synthesis of antimicrobial peptides, tryptophan-derived indole metabolites might reduce intestinal inflammatory damage and improve intestinal barrier integrity (51). Therefore, increased concentrations of *Lactobacillus* and *Bifidobacterium* might reduce obesity brought on by a high-fat diet. The *Akkermansia* genus had been reported to have the excellent ability to improve gut barrier function and reduce body weight (52). Several members of *Muribaculaceae* could lengthen the colon while lowering inflammatory cytokine levels, including IL-6, TNF-α, and IL-1β (53). One of the notable side effects of high-fat diet-induced obesity was the burst of inflammatory cytokines, directly related to energy management and metabolism (54). Inflammatory cytokines were also a significant contributor to the development of obesity-related comorbidities (55). Nevertheless, the relative abundance of *Muribaculaceae* was obviously raised by LBLF ingestion. Researchers found that decreased lipid levels in the serous and hepatic tissues were strongly connected with elevated *parasutterella* levels (56), although the precise mechanism is yet unknown. Short-chain fatty acid manufacturers including *Lactobacillus*, *Bifidobacterium*, and *Bacteroides* genera have been found to inhibit obesity and control the transcription and expression of enzymes involved in lipids metabolism (57, 58). Overall, some of the gut microbes that contribute to the mitigation of obesity and its related metabolic changes are selectively enriched after LBLF intervention.

An earlier study revealed that consuming a high-fat diet was directly related to the enrichment of the *Alistipes* genus in mice models and humans (59). Yang et al. (60) also found that enrichment of several members of *Alistipes* genus seriously impaired gut barrier function. Additionally, according to Gao et al. (61), the enrichment of the proinflammatory bacteria of the *Alistipes* genus might cause hepatic inflammation by managing mRNA expression of intestinal tight junctions proteins such as ZO-1, occludin that would then increase intestinal permeability. One of the specific microorganisms in the HFD group and a role in the serum rise of TC and TG were said to be the *Turicibacter* genus (62). Several microbial species from the *Lachnoclostridium* genus have been identified as possible colorectal cancer biomarkers (63). The transfer of lipopolysaccharide, one of the primary causes of obesity-related disorders, into the blood from the digestive tract may help some members of the *Lachnospiraceae* family to promote the development of insulin resistance and obesity (64). After LBLF administration, several gut bacteria that encourage obesity and its associated metabolic alterations were selectively decreased. Overall, it is predicted and stated that these gut bacteria might have a role in preventing or promoting obesity: 1) A long-term high-fat diet could increase intestinal permeability and damage gut barrier function in the host (65) as well as enhance relative contents of gut pathogenic bacteria such as *Alistipes*, *Lachnoclostridium*, etc. 2) Bacterial endotoxins, such as lipopolysaccharide, may be produced by gut pathogenic bacteria, leading to the formation of inflammation and oxidative stress (66). Additionally, the movement of endotoxin from the digestive tract into the blood circulation system was facilitated by impairing gut barrier function. 3) In response to the LBLF, some of the gut microbes such as *Akkermansia*, *Lactobacillus*, *Bifidobacterium*, etc might act as potential intestinal probiotics. These microbes may improve intestinal barrier function, relieve oxidative stress, inhibit the production of inflammatory factors, participate in cellular biochemical processes, and promote lipids metabolism via bacteria-mediated metabolites.

In conclusion, LBLF could prominently inhibit the development of high-fat diet-associated obesity and its related metabolic alterations with the reduction of body weight increase, Lee’s index, serum lipids, blood glucose, hepatic lipids, and oxidative stress levels, and increase of fecal lipids contents. Furthermore, LBLF increased gut bacterial community diversities, and the relative contents of beneficial bacteria while decreasing pathogenic bacteria. Taken together, the LBLF may offer a secure and innovative method for preventing foodborne obesity.

## Data availability statement

The sequences data presented in this study are deposited in the NCBI Sequence Read Archive (SRA) repository, accession number: PRJNA855047.

## Ethics statement

The animal study was reviewed and approved by Animal Welfare and Ethics Committee of Ningxia Medical University, Yinchuan, China.

## Author contributions

JL designed the experimental scheme, performed the experiment operation, analyzed the data, and finished the manuscript. YF guided and supervised the whole experimental process. JG participated in partial experiments. YN, TF, and FW gave some constructive advice on experimental design and revision of the manuscript. All authors contributed to the article and approved the submitted version.

## Conflict of interest

FW was employed by Ningxia Red Power Goji Co., Ltd. The remaining authors declare that the research was conducted in the absence of any commercial or financial relationships that could be construed as a potential conflict of interest.

## Publisher’s note

All claims expressed in this article are solely those of the authors and do not necessarily represent those of their affiliated organizations, or those of the publisher, the editors and the reviewers. Any product that may be evaluated in this article, or claim that may be made by its manufacturer, is not guaranteed or endorsed by the publisher.
